# Cysteine dioxygenase type 1: A new player in adipose physiology and metabolic health

**DOI:** 10.1016/j.ebiom.2022.104332

**Published:** 2022-11-03

**Authors:** Sardar Sindhu, Fatema Al-Rashed, Rasheed Ahmad

**Affiliations:** a Department of Immunology and Microbiology, Dasman Diabetes Institute, P.O.Box 1180, Dasman 15462, Kuwait; b Animal & Imaging Core Facilities, Dasman Diabetes Institute, P.O.Box 1180, Dasman 15462, Kuwait

Commentary for: “Adipose tissue *cysteine dioxygenase type 1* is associated with an anti-inflammatory profile, impacting on systemic metabolic traits”

Obesity is growing at epidemic proportions and looms as an alarming menace to global human health and wellbeing. This metabolic condition foreruns a constellation of metabolic dysfunctions and ensuing complications, such as hyperglycaemia, dyslipidaemia, insulin resistance, type-2 diabetes, cardiovascular disease, atherosclerosis, hypertension, neuropathy, and metabolic syndrome. The long-term risks involved are multifaceted and call for emerging needs to find novel targets of prognostic or therapeutic value.

Taurine, also known as 2-Aminoethanesulfonic acid, is a highly abundant, sulphur-containing, free amino acid found in mammals and it can be obtained exogenously from diverse dietary sources, such as meat, dairy products, fish, and shellfish as well as it is produced endogenously by the key rate limiting enzyme cysteine dioxygenase type 1 (CDO1) through *de novo* biosynthesis from cysteine and methionine substrates in the white adipose tissue, liver, and kidney.^1^ Taurine is pivotal in maintaining cellular and physiological homeostasis. These regulatory activities relate to glucose/lipid metabolism, adipogenesis, mitochondrial function, browning, thermogenesis, osmoregulation, redox/Ca^++^ balance, and reduced inflammatory and immune responses. There is now a growing consensus that obesity and type-2 diabetes are the taurine-deficient states.

Notably, lower plasma levels of taurine have been identified in humans with obesity/diabetes^2^ as well as in high fat diet (HFD)-induced or genetically obese mice.^3^ Whereas most studies underscoring the anti-obesity effects of taurine relate to animal work,^4^, ^5^, ^6^ there is only a handful of studies to substantiate alleviative effects of taurine in metabolic syndromes in humans,^7^^,^^8^ and perhaps none so far that captures modulations in CDO1 expression and activity in humans with obesity.

As though CDO1 expression and activity are highest in the liver, *CDO1* mRNA is also highly expressed in rodent adipose tissues and adipocytes.^1^^,^^9^ Adipose tissues play a central role in the pathogenesis of obesity and the expression changes and role of CDO1 in the subcutaneous (SAT) and visceral (VAT) white adipose tissue and the translational significance thereof in humans with obesity remain unclear. In an elegant study published in a recent issue of *eBiomedicine,* Latorre et al. investigated the *CDO1* gene expression levels and their associations with systemic metabolic profiles in a large Caucasian population comprising of 4 independent cohorts (N _Cohort 1_ = 299; N _Cohort 2_ = 150; N _Cohort 3_ = 65; and N _Cohort 4_ = 30 individuals) with varying obesity levels (Body mass index/BMI _Cohorts1–3_ = 20–68 kg/m^2^; BMI _Cohort 4_ >35 kg/m^2^).^10^ The clinical findings were further substantiated by performing a series of *in vitro* experiments involving a variety of cell types, such as isolated human adipocytes, human preadipocytes, and human immortalized adipose-derived mesenchymal stem cells (ACS52telo cells). Adipose samples from cohort 4 were also subjected to a high throughput RNAseq analysis.

Interestingly, the authors found that SAT/VAT *CDO1* transcripts expression was associated negatively with age, glycated haemoglobin (HbA1c), and fasting triglyceride (TG) levels, but positively with adipose gene expression of adipogenesis/thermogenesis/catabolism-related markers including adiponectin (*ADIPOQ*), fatty acid synthase (*FASN*), perilipin (*PLIN*)-*1*, solute carrier family 2 member A4 (*SLC2A4*), PR domain containing 16 (*PRDM16*), peroxisome proliferator-activated receptor-gamma coactivator-1 alpha (*PPARGC1A*), and cell death inducing DFFA like effector A (*CIDEA*) (Fig. 1). Multiple linear regression analysis identified *CDO1* as a contributory factor to reduced HbA1c/fasting TG levels and increased levels of high-density lipoprotein cholesterol (HDL-c) and adipose physiology markers referred to earlier. The reduced adipose *CDO1* expression was positively associated with increased intracellular TG accumulation and adipocyte hypertrophy. Overall, the findings obtained from cohort 1 were further validated using samples from cohorts 2 and 3.

**Fig. 1:**
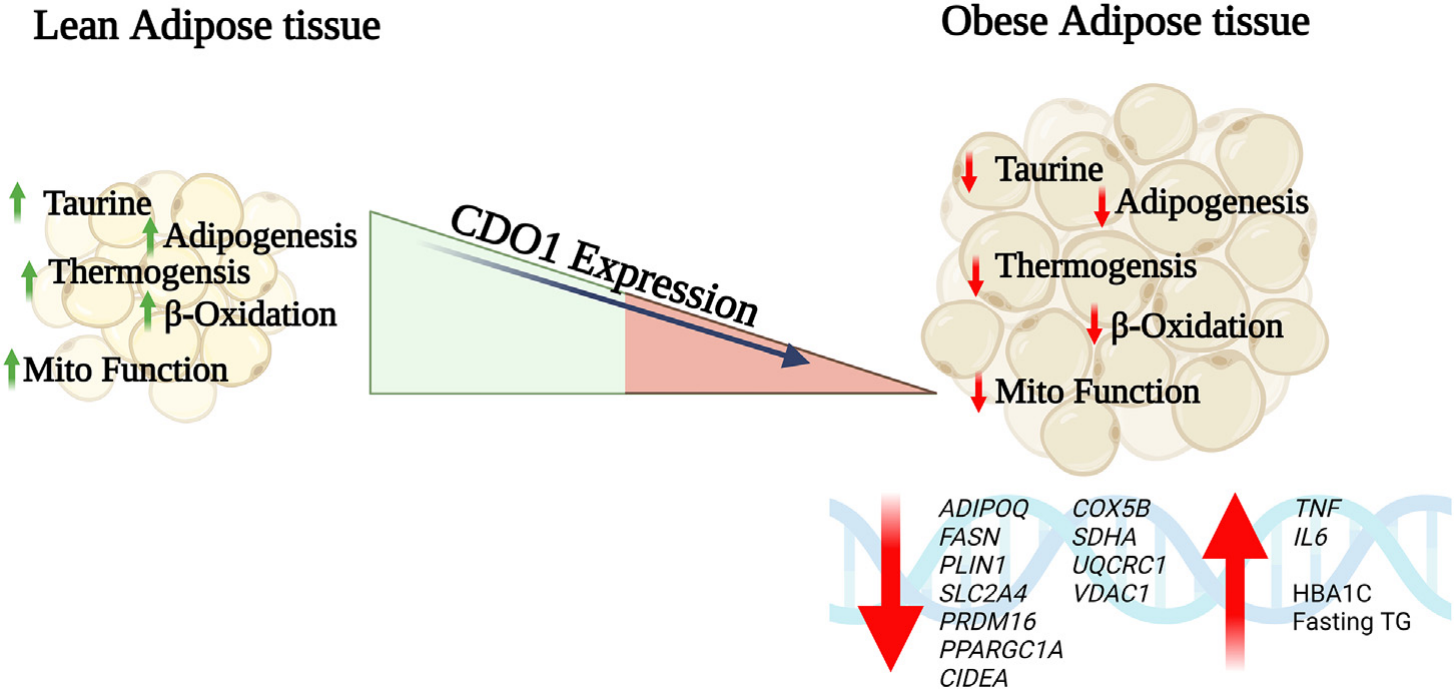
This illustration shows that in lean individuals, adipose *CDO1* expression regulates normal taurine biosynthesis, adipogenesis, thermogenesis, and mitochondrial function. However, in obesity, adipose *CDO1* expression is downmodulated, and as a result, markers of metabolic pathways related to adipogenesis, thermogenesis, energy expenditure, mitochondrial respiration and function are dysregulated. These changes also associate with increased inflammatory responses in the adipose tissue as well as hyperglycaemia and hypertriglyceridemia. This illustration was created with BioRender.com*.*

Relevance of CDO1 to the human adipose tissue biology and pathophysiology was verified through RNAseq analysis in cohort 4, wherein *CDO1* adipose expression (especially in VAT) was found to associate positively with critical regulatory pathways, such as fatty acid metabolism, glycolipid metabolism, insulin signalling/action, peroxisome proliferator-activated receptor (PPAR) signalling, AMP-activated protein kinase (AMPK) signalling, thermogenesis, adipocytokines, oxidative phosphorylation, mitochondrial activity and function (Fig. 1). Notably, *CDO1* expression was associated inversely with the pathways of inflammation, immune response, and cellular senescence.

*CDO1* expression was predominantly high in isolated adipocytes compared with stromal vascular fraction (SVF), and *in vitro* experimental data further showed the highest *CDO1* expression in fully differentiated human preadipocytes and ACS52telo cells. Inflammatory stimulus (LPS) reduced the *CDO1*/adipogenic genes, while the *CDO1* knockdown induced a proinflammatory effect (upregulated *TNF* and *IL6*) and led to a lower taurine biosynthesis. Furthermore, *CDO1* knockdown did not impact the mitochondrial biogenesis (unaltered *PPARGC1A*), but it did impair mitochondrial respiration/function (reduced *COX5B*, *SDHA*, *UQCRC1*, and *VDAC1*) as would be expected from taurine deficiency-mediated effects on mitochondrial biology and function.

This study has certain caveats. First, the data obtained represent only the Caucasian population which may raise concerns as to the lack of ethnic diversity in data and thus limited generalizability of the findings. Next, there are also no proof-of-concept *in vivo* data presented to support that adipose-specific CDO1 induction could improve the adipose dysfunction in HFD-induced or genetically obese mice. Notwithstanding, this remarkable work contributes the pioneering data from human SAT and VAT samples, underscoring CDO1 as a novel player in maintaining normal adipose physiology and function. Moreover, RNAseq data/pathway analyses substantiate the *in vitro* experimental data. The study also has translational significance, implying that enhancing the endogenous taurine biosynthesis via increased *CDO1* adipose expression/activity might be a more desirable and effective approach for restoring adipose function and metabolic health than administering taurine exogenously, especially as the taurine doses used in animal and human studies have rendered variable data.

Further extrapolating along these important findings, future studies should assess obesity-related changes in another major rate-limiting enzyme called cysteine sulfinic acid decarboxylase (CSAD), which is also linked to taurine biosynthesis in fat by catalysing decarboxylation of cysteine sulfinate to hypotaurine biosynthesis and evaluate its effects on adipose function and metabolic profiles in humans.

## Contributors

Literature search: S.S., F.A. and R.A.; figure preparation: F.A.; writing: S.S. and R.A. All the authors read and approved the final manuscript.

## Declaration of interests

The authors declare no conflict of interest.
